# Risk factors for placenta accreta spectrum: findings from the Japan environment and Children’s study

**DOI:** 10.1186/s12884-019-2608-9

**Published:** 2019-11-27

**Authors:** Hyo Kyozuka, Akiko Yamaguchi, Daisuke Suzuki, Keiya Fujimori, Mitsuaki Hosoya, Seiji Yasumura, Tadahiko Yokoyama, Akiko Sato, Koichi Hashimoto, the Japan Environment and Children’s Study (JECS) Group, Toshihiro Kawamoto, Hirohisa Saito, Reiko Kishi, Nobuo Yaegashi, Koichi Hashimoto, Chisato Mori, Shuichi Ito, Zentaro Yamagata, Hidekuni Inadera, Michihiro Kamijima, Takeo Nakayama, Hiroyasu Iso, Masayuki Shima, Yasuaki Hirooka, Narufumi Suganuma, Koichi Kusuhara, Takahiko Katoh

**Affiliations:** 1 Fukushima Regional Center for the Japan Environmental and Children’s Study, 1, Hikarigaoka, Fukushima, 960-1295 Japan; 2 Department of Obstetrics and Gynecology, School of Medicine, Fukushima Medical University, 1, Hikarigaoka, Fukushima, 960-1295 Japan; 3 Department of Pediatrics, School of Medicine, Fukushima Medical University, 1, Hikarigaoka, Fukushima, 960-1295 Japan; 4 Department of Public Health, School of Medicine, Fukushima Medical University, Fukushima, Japan, 1, Hikarigaoka, Fukushima, 960-1295 Japan; 5 Fukushima medical center for women and children, Fukushima medical university school of medicine, 1, Hikarigaoka, Fukushima, 960-1295 Japan

**Keywords:** Placenta accreta spectrum, Placenta previa, Birth cohort study, Smoking, Uterine anomaly, Assisted reproductive technology

## Abstract

**Background:**

Placenta accreta spectrum (PAS) is a life-threating complication in the field of obstetrics. Sometimes we face with unexpected PAS cases which is potentially higher maternal mortality and morbidity compared with expected cases. The present study was conducted to examine the prevalence of PAS and to elucidate its risk factors using a large Japanese birth cohort study.

**Methods:**

We reviewed the results of a nationwide prospective birth cohort study in Japan, and identified 90,554 participants treated from 2011 to 2014 in 15 regional centers. Multiple regression models were created to identify the risk factors for PAS. These data were obtained from self-reported questionnaires or patient medical records.

**Results:**

This analysis consisted of 202 cases of PAS (18 with placenta previa and 184 without placenta previa) and 90,352 cases without PAS. The multiple logistic regression analysis showed that placenta previa (adjusted odds ratio [aOR]: 12.86, 95% confidence interval [CI] 7.70–21.45, *P* < 0.001), assisted reproductive technology-related pregnancies (aOR: 6.78, 95% CI 4.54–10.14, *P* < 0.001), smoking during pregnancy (aOR: 1.95, 95% CI 1.15–3.31, *P* = 0.013), more than two previous cesarean sections (aOR: 2.51, 95% CI 1.35–4.67, *P* = 0.004), and uterine anomalies (aOR: 3.97, 95% CI 1.24–12.68, *P* = 0.020) increased the risk of PAS.

**Conclusion:**

In general population, placenta previa, assisted reproductive technology-related pregnancy, smoking during pregnancy, repeated cesarean sections, and uterine anomalies were risk factors for PAS in the Japanese population.

## Background

Placenta accreta spectrum (PAS) is a significant obstetric complication that can cause massive and life-threatening bleeding. It is widely recognized that previous cesarean sections (CS) and placenta previa are risk factors for abnormal placentation [1]. With the increase in the CS birth rate, the incidence of PAS has increased [2]. Wu et al. reported that the incidence of placenta accreta was 1 of 533 births in 1982–2002 [3], which is considerably higher than the incidence reported in previous studies, ranging from 1 of 4027 births to 1 of 2510 births in the 1970s to 1980s [2, 4]. This condition could increase maternal morbidities, such as hemorrhage (adjusted odds ratio [aOR]: 16.6, 95% confidence interval [CI]: 13.4–20.5), transfusion (aOR: 41.8, 95% CI: 33.4–52.2), and hysterectomy (aOR: 950, 95% CI: 632.9–1427.9) [5].

The association between PAS and current or previous placenta previa suggests that the possibility of placental adhesions may be a factor in the development of PAS. Placental adhesions are thought to be caused by the placenta adhering to a defective site in the decidua [6]. In such cases, careful diagnosis and multidisciplinary management strategies are required before CS to reduce the risk of morbidity [7]. However, on occasion, we have been faced with an unexpected PAS in the absence of placenta previa that was diagnosed for the first time after delivery. Most of these patients were clinically diagnosed as having PAS and these situations required unexpected medical intervention, including manual removal of the adherent placenta, which could have caused life-threatening postpartum hemorrhage [8] and/or increased morbidity [9]. Therefore, it is crucial to maintain a high index of suspicion for PAS in the antenatal period in high-risk patients without placenta previa.

Several studies and case reports have tried to identify specific risk factors for abnormal placental invasion, other than placenta previa or repeated CS. However, these studies were limited by small numbers of placenta accreta cases or retrospective study designs. Hence, the aim of this study was to examine the prevalence of PAS and to identify its risk factors by evaluating data from the largest prospective birth cohort study in the Japanese population.

## Methods

In this study, we investigated the results of the Japan Environment and Children’s Study (JECS), which is a nationwide, government-funded, birth cohort study [10] that was started in January 2011 to investigate the effects of environmental factors on children’s health. The eligibility criteria for the JECS participants (expectant mothers) were as follows: (1) residing in one of the study areas at the time of recruitment and expected to reside continually in Japan for the foreseeable future, (2) an expected delivery date between August 1, 2011 and mid-2014, and (3) the ability to participate in the study without difficulty (i.e., the participant needed to be able to comprehend the Japanese language and complete the self-administered questionnaires). This study was conducted in 15 regional centers across Japan as described previously [10].

### Data collection

Data for this analysis utilized the JECS dataset released in June 2016 (dataset: jecs-ag-20,160,424). We used two types of data: (1) T1: comprising data obtained from self-reported questionnaires collected around the participants’ first trimesters (the first questionnaire), and including questions related to the maternal medical background; (2) M0: obstetrics outcome collected from medical records provided by each participant’s institution. Data of participants with multiple-gestation pregnancies and those with insufficient data were excluded from the analysis.

### Maternal medical background

The maternal medical background information was obtained from the M0 data (maternal age at time of delivery, pre-pregnancy body mass index [BMI], and parity), T1 data (maternal smoking status, number of previous induced abortions [IA], number of previous CSs, manner of conception, and pre-pregnancy gynecological complications, including endometriosis, uterine myomas, adenomyosis, and uterine anomalies). The mothers were categorized into six age groups: < 20, 20–24, 25–29, 30–34, 35–39, and ≥ 40 years. The BMIs were calculated according to World Health Organization standards (body weight [kg]/height^2^ [m^2^]). We further categorized the participants into three groups according to their BMI: < 18.5, 18.5–25.0, and ≥ 25.0 kg/m^2^. The manner of conception was categorized as natural or assisted reproductive technology (ART)-related, with ART defined as conception after in vitro fertilization (IVF) and/or intracytoplasmic sperm injection (ICSI), or cryopreserved, frozen, or blastocyst embryo transfers. Maternal participants were requested to provide information about their smoking status, which was categorized using the following statements: “kept smoking during pregnancy,” “quit smoking during early pregnancy,” “never smoked,” and “quit smoking before pregnancy.” We classified maternal smoking into three categories. The maternal participants who chose “kept smoking during pregnancy” were classified as “smoking during pregnancy.” The maternal participants who chose “quit smoking during early pregnancy” and “quit smoking before pregnancy or never smoked” were classified as “quit during early pregnancy” and “never smoker,” respectively. The numbers of previous IAs and CSs were categorized into three groups: 0, 1, and ≥ 2. Maternal participants were also asked to answer the question: “Have you ever been diagnosed as having a uterine anomaly (or other pre-pregnancy gynecological condition, i.e., endometriosis, uterine myoma, and adenomyosis) in a medical institution?” The maternal participants who answered “yes” were classified as having a uterine anomaly (or other pre-pregnancy gynecological condition). The pre-pregnancy gynecological complications obtained from the self-reported questionnaire of JECS were validated previously [11, 12].

### Obstetrical outcomes

Obstetrical outcomes were obtained from the M0 data and included the following: gestational age at the time of delivery, the presence or absence of placenta previa, presence or absence of PAS, mode of delivery, and maternal transfusion. The mode of delivery was categorized into vaginal delivery or CS. In the present study, the definition of PAS was dependent on the obstetrician in charge, but its diagnosis was based on results of the histological examination or the clinical presentation, as follows: 1) difficulty in manual removal of the placenta partially or totally, and no evidence of placental separation from the uterus, despite management; 2) sonographic evidence of retained placental fragments requiring curettage; and 3) heavy bleeding from the implantation site after manual removal of the placenta by the attending obstetrician at the time of delivery.

### Statistical analysis

The data of the women with PAS were reviewed. The frequency of PAS with or without placenta previa was explored according to the number of previous CSs. Next, the data were categorized into two groups: those who had indications of the presence of PAS, and those who had indications of the absence of PAS. Maternal medical backgrounds and obstetrical outcomes were compared between the two groups. The chi-square or Fisher exact test was used to compare the categorical variables, and the t-test was used to compare the continuous variables after confirming each of the continuous variables was normally distributed. The extended Mantel-Haenszel chi-square test of linear trends was used to analyze proportional trends. The aOR and 95% CI for PAS were calculated using a multiple logistic regression model. The ORs were adjusted for placenta previa, ART, number of previous CSs, uterine anomalies, adenomyosis, endometriosis, uterine myomas, maternal age, and number of previous IAs. In the logistic regression model, dummy variables were used for categorical variables that consisted of more than three categories. SPSS version 21 (IBM Corp., Armonk, NY) was used for the statistical analyses. The level of statistical significance was set at *P* < 0.05.

## Results

**Fig. 1:**
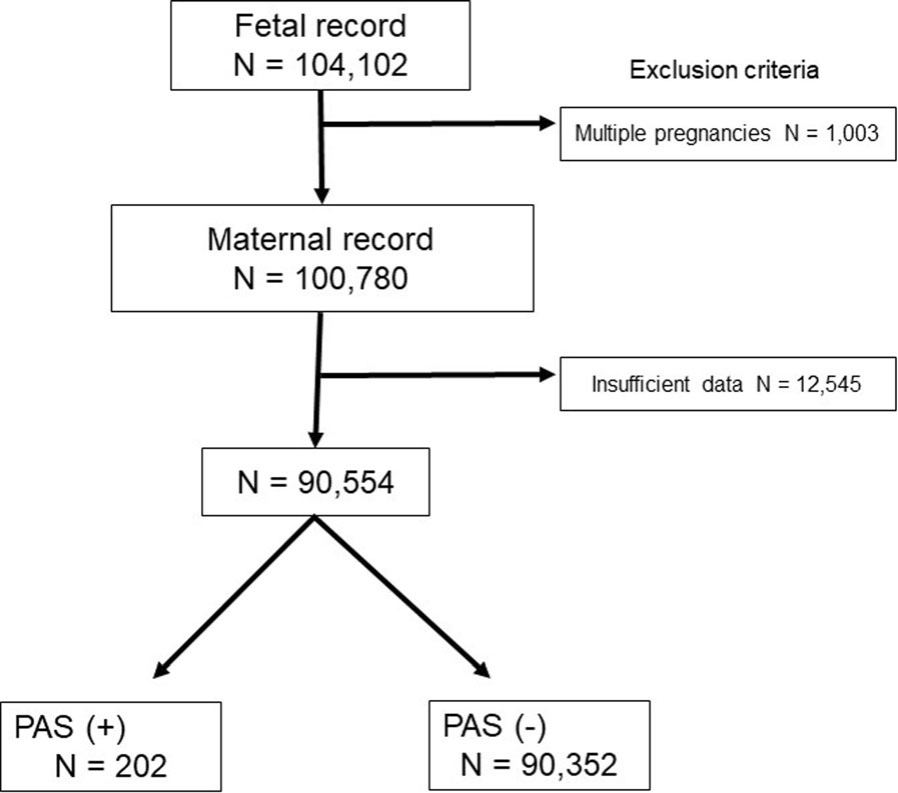
Enrollment and inclusion in analysis. PAS: placenta accreta spectrum

There were 104,102 records identified during the study period. Of those, 1003 records from women with multiple gestation pregnancies and 12,545 maternal participants with insufficient data were excluded from the analysis (Fig. 1). After applying our exclusion criteria, 90,554 maternal participants were eligible for the analysis, comprising 202 women with PAS (18 with placenta previa and 184 without placenta previa) and 90,352 women without PAS. The prevalence rates of placenta previa and PAS in this study were 0.6% (531/90,554) and 0.2% (202/90,554), respectively.

Table 1 summarizes the basic characteristics of the participants according to the presence or absence of PAS. Both the mean maternal ages and the maternal age categories were significantly different between the two groups (*P* < 0.001 and *P* = 0.001, respectively). There were no significant differences in either the number of previous IAs or BMI between the two groups (*P* = 0.468 and *P* = 0.925, respectively). The proportion of ART-related pregnancies was 19.3% and the rate of smoking during pregnancy was 8.4% among the patients with PAS, which was significantly higher than the rates among those without PAS (*P* < 0.001 and *P* = 0.029, respectively). There were no significant differences between patients with and without PAS with respect to primiparity (37.6 and 40.6%, respectively; *P* = 0.397), gestational age (38.7 [2.1] weeks and 38.7 [2.1] weeks, respectively; *P* = 0.964), or CS (16.8 and 19.3%, respectively; *P* = 0.378). Some pre-pregnancy gynecological complications, although not significant, were more often seen in patients with PAS than in those without, including endometriosis (5.9 and 3.7%, respectively; *P* = 0.084), uterine myomas (8.9 and 6.1%, respectively; *P* = 0.090), and adenomyosis (1.0 and 0.3%, respectively; *P* = 0.099). However, uterine anomalies were significantly more common in patients with PAS than in those without PAS (1.5% versus [vs.] 0.3%; *P* = 0.021). The rate of maternal blood transfusion was significantly higher in patients with PAS than in those without PAS (19.3% vs. 0.4%; *P* < 0.001).

**Table 1:** Basic characteristics of the participants according to the presence or absence of placenta accreta spectrum

Variable	PAS (+) *N* = 202	PAS (−) *N* = 90,352	*P* value
Maternal age, mean years (SD)	32.4 (5.3)	31.2 (5.0)	< 0.001^a^
Maternal age category, % (n)			
≤ 19 years	1.0 (2)	0.8 (743)	0.001^b^
20–24 years	6.4 (13)	9.1 (8192)	
25–29 years	21.8 (44)	27.7 (25011)	
30–34 years	32.2 (65)	35.4 (32025)	
35–39 years	29.7 (60)	22.4 (20274)	
≥ 40 years	8.9 (18)	4.5 (4107)	
Primiparous, % (n)	37.6 (76)	40.6 (36643)	0.397^b^
ART pregnancy, % (n)	19.3 (39)	2.9 (2619)	< 0.001^b^
Maternal smoking status, % (n)			
Never smoker	81.7 (165)	82.2 (74294)	0.029^b^
Quit smoking during early pregnancy	9.9 (20)	13.0 (11742)	
Smoking during pregnancy	8.4 (17)	4.8 (4316)	
Number of IAs, % (n)			
0	81.2 (164)	84.2 (76073)	0.468^b^
1	13.4 (27)	11.6 (10506)	
≥ 2	5.4 (11)	4.2 (3773)	
BMI, kg/m^2^, % (n)			
< 18.5	16.2 (32)	16.2 (14471)	0.925^b^
18.5–24.9	72.2 (143)	73.1 (65362)	
≥ 25	11.6 (23)	10.8 (9618)	
Endometriosis, % (n)	5.9 (12)	3.7 (3301)	0.084^b^
Uterine myoma, % (n)	8.9 (18)	6.1 (5475)	0.090^b^
Adenomyosis, % (n)	1.0 (2)	0.3 (295)	0.099^c^
Uterine anomaly, % (n)	1.5 (3)	0.3 (256)	0.021^c^
Gestational age, mean weeks (SD)	38.7 (2.1)	38.7 (2.1)	0.964^a^
Placenta previa, % (n)	8.9 (18)	0.6 (513)	< 0.001^b^
Cesarean section, % (n)	16.8 (34)	19.3 (17301)	0.378^b^
Maternal blood transfusion, % (n)	19.3 (39)	0.4 (416)	< 0.001^b^

*PAS* Placenta accreta spectrum, *SD* Standard deviation, *ART* Assisted reproductive technology, *IA* Induced abortion, *BMI* Body mass index

^a^ *P* value from t-test

^b^ *P* value from chi-square test

^c^ *P* value from Fisher exact test

*P* < 0.05 is statistically significant

Table 2 summarizes the association between the number of previous CSs and the incidence of PAS with respect to the presence or absence of placenta previa. In patients with placenta previa, the incidence of PAS was significantly increased at 1.3% (6/460), 12.2% (6/49), and 27.3% (6/22) for 0, 1, and ≥ 2 previous CSs (*P* < 0.001). In patients without placenta previa, the incidences of PAS were 0.2% (173/81,914), 0.1% (7/6081), and 0.2% (5/2028) for 0, 1, and ≥ 2 previous CSs, which were not significantly different (*P* = 0.427).

**Table 2:** Frequency of placenta accreta spectrum according to the number of previous cesarean sections

Number of previous CSs	Placenta previa (*P* < 0.001*)	No placenta previa (*P* = 0.427*)
0	1.3% (6/460)	0.2% (172/81914)
1	12.2% (6/49)	0.1% (7/6081)
≥2	27.3% (6/22)	0.2% (5/2028)

*CS* Cesarean section

*, *P* value from the extended Mantel-Haenszel chi-square test

*P* < 0.05 is statistically significant

Table 3 shows the results of the logistic regression analyses. After controlling for potential risk factors, placenta previa (aOR: 12.86, 95% CI 7.70–21.45; *P* < 0.001), ART (aOR: 6.78, 95% CI 4.54–10.14; *P* < 0.001), smoking during pregnancy (aOR: 1.95, 95% CI 1.15–3.31; *P* = 0.013), ≥2 previous CSs (aOR: 2.51, 95% CI 1.35–4.67; *P* = 0.004), and uterine anomalies (aOR: 3.97, 95% CI 1.24–12.68; *P* = 0.020) were all related to PAS.

**Table 3:** Factors associated with placenta accreta spectrum: results from univariate and logistic regression analyses

Variable	Univariate analysis			Multivariate analysis		
	OR	95% CI	*P* value	aOR	95% CI	*P* value
Placenta previa	17.13	10.48–28.01	< 0.001	12.86	7.70–21.45	< 0.001
ART pregnancy	8.02	5.64–11.39	< 0.001	6.78	4.54–10.14	< 0.001
Maternal smoking status						
Never smoker	Ref	–	–	Ref	–	–
Quit smoking during early pregnancy	0.74	0.46–1.17	0.193	0.83	0.51–1.35	0.452
Smoking during pregnancy	1.83	1.11–3.01	0.017	1.95	1.15–3.31	0.013
Number of previous CSs						
0	Ref	–	–	Ref	–	–
1	0.95	0.54–1.66	0.850	0.87	0.49–1.53	0.618
≥ 2	2.49	1.36–4.59	0.003	2.51	1.35–4.67	0.004
Uterine anomaly	5.31	1.69–16.70	0.004	3.97	1.24–12.68	0.020
Adenomyosis	3.05	0.76–12.35	0.118	1.77	0.41–7.52	0.442
Endometriosis	1.67	0.93–2.99	0.087	0.81	0.42–1.55	0.525
Uterine myoma	1.52	0.93–2.46	0.092	1.02	0.61–1.69	0.951
Maternal age						
≤ 19 years	1.21	0.30–4.87	0.792	1.68	0.38–7.48	0.494
20–24 years	Ref	–	–	Ref	–	–
25–29 years	0.73	0.52–1.02	0.062	1.05	0.56–1.96	0.878
30–34 years	0.86	0.64–1.16	0.333	1.06	0.58–1.95	0.848
35–39 years	1.46	1.08–1.98	0.014	1.23	0.66–2.30	0.516
≥ 40 years	2.05	1.27–3.34	0.004	1.41	0.66–3.01	0.373
Number of IAs						
0	Ref	–	–	Ref	–	–
1	1.17	0.78–1.76	0.442	1.30	0.85–1.97	0.224
≥ 2	1.32	0.72–2.43	0.369	1.29	0.68–2.45	0.433

*ART* Assisted reproductive technology, *IA* Induced abortion, *OR* Odds ratio, *CI* Confidence interval, *aOR* Adjusted odds ratio, *Ref* Reference, *CS* Cesarean section

## Discussion

To the best of our knowledge, this is first study that reports the prevalence of and risk factors for PAS based on the data of a large cohort study in Japan. Although the risk factors for PAS identified in this study, including placenta previa, ART-related pregnancy, smoking during pregnancy, and repeated CS, are compatible with those of previous studies [1, 13–15], this study is the first to also identify uterine anomalies as a risk factor for PAS after accounting for several risk factors.

In the present study, the incidence of PAS was 222/100,000 births, which is in accordance with a previous review of 34 studies that reported an average incidence of 189/100,000 births [16]. This study confirmed the association between previous numbers of CSs and PAS in women with placenta previa. This study’s finding is consistent with those of previous studies conducted in the United States [1]. However, PAS occurred in only 27.3% of the women with placenta previa who experienced ≥2 CSs, substantially less than the prevalence of 48.2% that was reported previously, which might result from the different study design methodologies and diagnostic criteria for abnormal placenta invasion.

Our results suggest that smoking during the first trimester, but not at the time of conception, increases the risk of PAS. The mechanism as to why smoking during pregnancy is related to abnormal placentation is unknown. One scenario proposed by Michikawa et al. is that systemic inflammation induced by air pollutants [17, 18] affects the uterine endometrium, leading to poor decidualization [19]. An animal experiment also reported that exposure to fine particulate matter (PM_2.5_), an air pollutant, during pregnancy was related to placental inflammation [20]. Therefore, it is reasonable to assume that pollutant-induced inflammation during pregnancy could also cause inflammation in the endometrium [21], resulting in placental adhesion to the uterus.

With regard to the association between ART pregnancy and PAS, our results are consistent with those of previous reports [22, 23]. The reason why ART pregnancies may be at an increased risk of placental adhesion is still unknown. Esh et al. proposed two possible pathogeneses: 1) mechanical factors (primary deficiency in the decidua due to local trauma at the uterine wall), and 2) biological factors (abnormal maternal response to trophoblast invasion) [23].

Congenital anomalies of the vagina, cervix, and uterus arise from errors in embryogenesis. Although there are several diversities in the forms of uterine anomaly, Mu¨llerian defects are associated with minimal obstetric risk and others are linked to significant morbidity, including first and second trimester losses, fetal growth restriction, malpresentation, and premature birth [24]. With respect to uterine anomalies as risk factors for PAS, most studies are restricted to case series. Oral et al. reported in their cases series that the prevalence of placenta accreta in rudimentary uterine horn pregnancies may be greater than 10% due to the thinness of the myometrium that easily led to placental invasion into the myometrium [25].

Identifying the risk factors for PAS is important because patients at risk would have an opportunity to be evaluated more carefully by screening for placenta accreta using 3-dimensional power Doppler and magnetic resonance imaging [26, 27]. Furthermore, women with suspected PAS would be recommended to deliver their infants in a tertiary care hospital with a multidisciplinary team available for managing the severe postpartum hemorrhage. The benefit to these patients would be significant because of the potential for decreasing mortality via multidisciplinary team management [6].

The strength of this study is that it is the first large-scale, nationwide, population-based study in Japan that investigated various factors in the evaluation of pregnant women with PAS. Therefore, this study is considered to be representative of the general pregnant population in Japan and relatively free of selection bias [28]. The prospective data were collected by physicians, midwives, nurses, and trained research coordinators, and therefore, are more likely to be accurate. As mentioned previously, this study presents clear definitions of smoking status during pregnancy. To the best of our knowledge, this is the first report presenting smoking as a risk factor for PAS based on a clear definition of smoking status as the potential variable.

This study also has some limitations. The most substantial limitation of this study is that although the diagnosis of PAS was based on medical records from each institution, we are not completely aware of the severity of invasion into the myometrium (accreta, increta, or percreta) and pathology reports were not required for the diagnosis of PAS. Therefore, most of PAS cases in this study were clinically diagnosed and is thought to be unexpected PAS cases which were not accompanied with placenta previa. This limitation has resulted in the identification on only 18 cases of true PAS which was in combination with placenta previa. However, our results indicate that the rate of maternal transfusion was substantially higher in the PAS group than in the non-PAS group. Further more recent evidence suggested that outcomes in the unexpected PAS cases, which was consisted of 35/54 (62.7%) cases without placenta previa, were poor than in antenatal diagnosed PAS cases, which was consisted of 135/189 (74.6%) with placenta previa [29]. Therefore, although most PAS cases in the present analysis consisted of unexpected PAS cases which was diagnosed clinically, we believe that it is worth identifying the risk of unexpected PAS is important from the view of maternal mortality and morbidity.

With respect to the maternal background data, we relied on a self-reported questionnaire instead of objective measurements of gynecological complications before pregnancy. As such, we were not aware of uterine anomaly patterns (i.e., unicornuate uterus, uterine didelphys, or bicornuate uterus) or a history of uterine surgery in each case. The specific ART methods (IVF and/or ICSI, cryopreserved, frozen or blastocyst embryo transfer) were not classified in this study. Although we accounted for several confounders in large portions of the questionnaire, unknown risk factors for PAS might have existed. We also did not include some of the risk factors previously reported, such as previous uterine surgeries [15] or hypertensive disorders [8].

## Conclusion

After adjusting for several confounding factors, we determined that ART-related pregnancy, smoking during pregnancy, and uterine anomalies are risk factors for PAS, in addition to the previously well-known risk factors of placenta previa and repeated CS.

## Data Availability

The datasets analyzed during the current study are not publicly available due to confidentiality/research subject protections. The authors, with permission of the Eco-child Study Investigation Committee and the Japan government, can make the datasets available upon reasonable request.

## Abbreviations

aOR: Adjusted odds ratio
BMI: Body mass index
CI: Confidence interval
CS: Cesarean sections
IA: Induced abortion
ICSI: Intracytoplasmic sperm injection
IVF: In vitro fertilization
JECS: Japan Environment and Children’s Study
PAS: Placenta accreta spectrum
vs: Versus
